# Deep learning accurately classifies elbow joint effusion in adult and pediatric radiographs

**DOI:** 10.1038/s41598-022-16154-x

**Published:** 2022-07-12

**Authors:** Jarno T. Huhtanen, Mikko Nyman, Dorin Doncenco, Maral Hamedian, Davis Kawalya, Leena Salminen, Roberto Blanco Sequeiros, Seppo K. Koskinen, Tomi K. Pudas, Sami Kajander, Pekka Niemi, Jussi Hirvonen, Hannu J. Aronen, Mojtaba Jafaritadi

**Affiliations:** 1 Faculty of Health and Well-Being, Turku University of Applied Sciences, Turku, Finland; 2 Department of Radiology, University of Turku, Turku, Finland; 3 Department of Radiology, University of Turku and Turku University Hospital, Turku, Finland; 4 Faculty of Engineering and Business, Turku University of Applied Sciences, Turku, Finland; 5 Department of Nursing Science, University of Turku and Director of Nursing (Part-Time) Turku University Hospital, Turku, Finland; 6 Terveystalo Inc, Jaakonkatu 3, Helsinki, Finland

**Keywords:** Diagnosis, Health services, Medical imaging, Paediatrics, Prognosis, Public health, Musculoskeletal system, Bone, Characterization and analytical techniques, Imaging techniques

## Abstract

Joint effusion due to elbow fractures are common among adults and children. Radiography is the most commonly used imaging procedure to diagnose elbow injuries. The purpose of the study was to investigate the diagnostic accuracy of deep convolutional neural network algorithms in joint effusion classification in pediatric and adult elbow radiographs. This retrospective study consisted of a total of 4423 radiographs in a 3-year period from 2017 to 2020. Data was randomly separated into training (n = 2672), validation (n = 892) and test set (n = 859). Two models using VGG16 as the base architecture were trained with either only lateral projection or with four projections (AP, LAT and Obliques). Three radiologists evaluated joint effusion separately on the test set. Accuracy, precision, recall, specificity, F1 measure, Cohen’s kappa, and two-sided 95% confidence intervals were calculated. Mean patient age was 34.4 years (1–98) and 47% were male patients. Trained deep learning framework showed an AUC of 0.951 (95% CI 0.946–0.955) and 0.906 (95% CI 0.89–0.91) for the lateral and four projection elbow joint images in the test set, respectively. Adult and pediatric patient groups separately showed an AUC of 0.966 and 0.924, respectively. Radiologists showed an average accuracy, sensitivity, specificity, precision, F1 score, and AUC of 92.8%, 91.7%, 93.6%, 91.07%, 91.4%, and 92.6%. There were no statistically significant differences between AUC's of the deep learning model and the radiologists (*p* value > 0.05). The model on the lateral dataset resulted in higher AUC compared to the model with four projection datasets. Using deep learning it is possible to achieve expert level diagnostic accuracy in elbow joint effusion classification in pediatric and adult radiographs. Deep learning used in this study can classify joint effusion in radiographs and can be used in image interpretation as an aid for radiologists.

## Introduction

Radiographs are still the first-choice imaging modality in elbow trauma^1^. In the adult and pediatric elbow there can be occult fractures that are not visible in radiographs^1,2^. Elbow fractures are easily missed especially in the pediatric population^3^ due to cartilaginous appearance in the elbow radiographs. In these cases, however, joint effusion can be seen in the lateral projection via displacement of anterior and/or posterior fat pads^2^. Fat pads are intracapsular but extrasynovial anatomical structures^4^ that can be classified either as positive (abnormal) or negative (normal) fat pads in radiographs. In normal radiographs with negative fat pads, only the anterior fat pad can be seen in contact with anterior humerus, while the posterior fat pad is hidden in the olecranon fossa. In the case of intracapsular fracture, the positive anterior fat pad is elevated, and is thereby more sensitive in showing joint effusion. A positive posterior fat pad is recognized by the displacement of the fat pad dorsally out of the olecranon fossa as a result of joint effusion^5^. Although the clinical relevance with joint effusion is debatable^6^, it is an important finding that should be in the radiology report. When joint effusion is noted without a visible fracture, given the cost-efficiency, follow-up is recommended^7^.

Errors in image interpretation can lead to worse patient outcomes^8^, and it is crucial that radiology profession is motivated in finding ways that artificial intelligence (AI) can improve patient treatment^9–11^, minimize image interpretation errors^10,12^ and improve the profession^11^. AI competence has been studied in various body parts^13–16^, in different non-traumatic conditions^17–20^, traumatic conditions^10,21–23^ and in comparison, with radiologists' level detection of abnormalities^24,25^. Deep convolutional neural networks (DCNN) can increase fracture detection^26^ support clinical decision making^12,27^. DCNN has been used to detect joint effusion from lateral elbow radiographs with a sensitivity of 0.91, a specificity of 0.91, and an accuracy of 0.91^28^. DCNN models are also able to detect supracondylar fractures comparable to radiologists^29,30^. However, to the best of our knowledge there are no studies utilizing DCNN in large clinical populations with both pediatric and adult patients.

The purpose of this study is to investigate DCNN accuracy in classification of joint effusion in pediatric and adult elbow radiographs. We hypothesized that (1) DCNN will accurately classify joint effusion in elbow radiographs including adult and pediatric patients, and (2) DCNN will reach accuracy levels of three expert radiologists. Transfer learning strategy was proposed where a pre-trained model was reused to train a new model on a different vision task. Transfer learning was used to train our neural network because comparatively little data is required for it to be efficient. This approach offers several advantages, such as reduction in training time and improved performance of neural networks. The new task considers using a pretrained convolutional neural network that receives elbow radiographs as inputs and outputs the probability of effusion along with a heatmap localizing the areas of the image most indicative of effusion.

## Materials and methods

### Data collection and annotation

**Figure 1:**
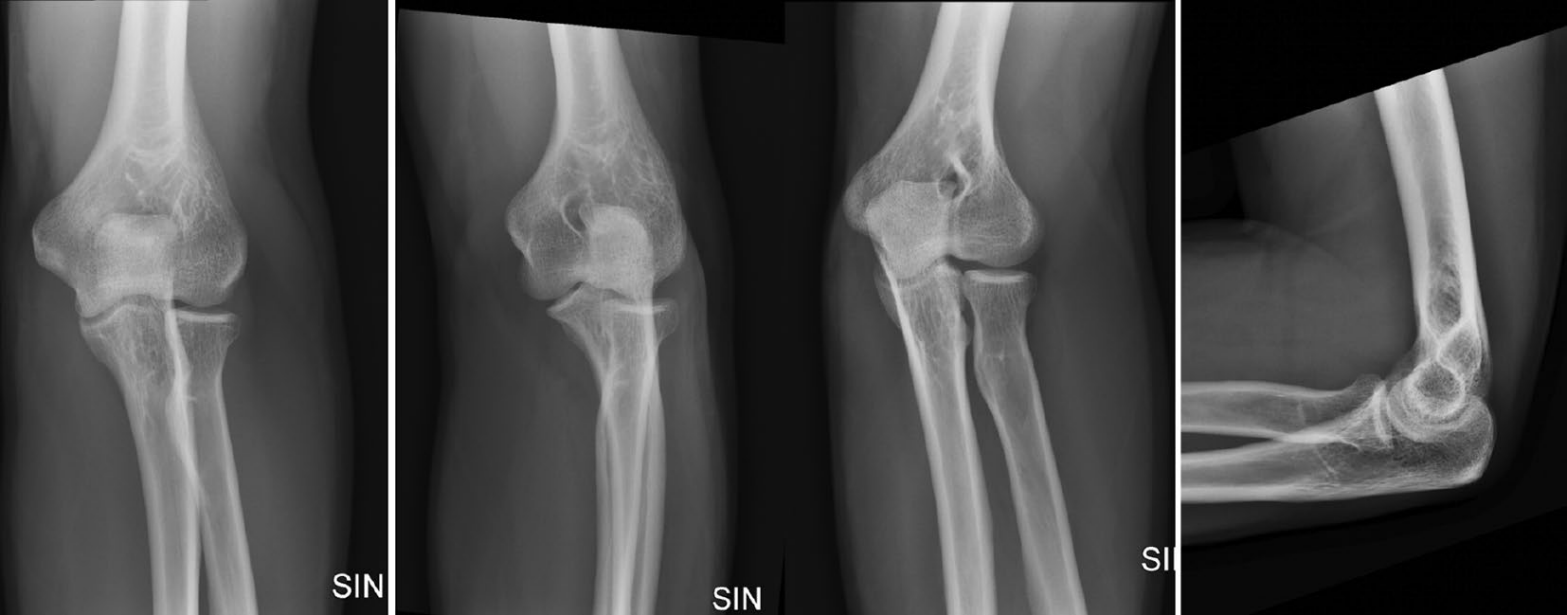
Example of normal adult radiographs including AP, external oblique, internal oblique and lateral projections used in this study.

This retrospective study received ethical approval from the Ethics Committee of the University of Turku (ETMK Dnro: 38/1801/2020). This study complies with the Declaration of Helsinki and was performed according to ethics committee approval. Because of the retrospective nature of the study, need for informed consent was waived by the Ethics Committee of the Hospital District of Southwest Finland. In this study 1309 elbow patient cases were collected from Turku University Central hospital’s picture archiving and communication system, including 634 cases of positive and 675 negative fatpad/effusion cases (Table 1), of which 208 cases were excluded due to various reason. Therefore, this study included 1101 elbow patient cases (de-identified) with a total of 4423 radiographs between 2017 and 2020 and associated radiology reports. Table 2 also shows patient's demographic information in different subsets. All radiographs were obtained with the same machinery (Carestream Evolution 2012, B-103H, United States) in an emergency radiology department in a tertiary care referral center. Images were obtained in Digital Imaging and Communications in Medicine (DICOM) format. AP and oblique projections were taken with 57 kVp and 4 mAs and lateral projection with 57 kVp and 5 mAs (Fig. 1). Inherent filtration was 3.17 mmAl with no added filtration.

**Table 1:** Patient demographics in pediatric and adults groups (from the main data registry).

Patients’s demographics	Positive fatpad/effusion (n = 634)	Negative fatpad/effusion (n = 675)	*p* value*
**Age (years)**			
Pediatrics (n = 490)	8.6 (1–18)	10.95 (0–18)	< 0.005
Adults (n = 819)	49.56 (19–97)	49.85 (19–98)	0.83
**Sex (pediatric)**			
Female	134 (121)	109 (126)	0.17
**Sex (adults)**			
Female	146 (233)	223 (217)	< 0.005

*Two-sample t-test significant test.

**Table 2:** Patient demographics in different subsets.

Patients’s Demographics	Training (n = 666)	Validation (n = 222)	Test (n = 213)	Total (N = 1101)
**Age (years)**				
Mean (whole population)	34.6	35.5	33	
Pediatric (1–18)	248	80	79	407
Adults (19–98)	418	142	134	694
**Sex**				
Male	324	101	91	516
Female	342	121	122	585

**Figure 2:**
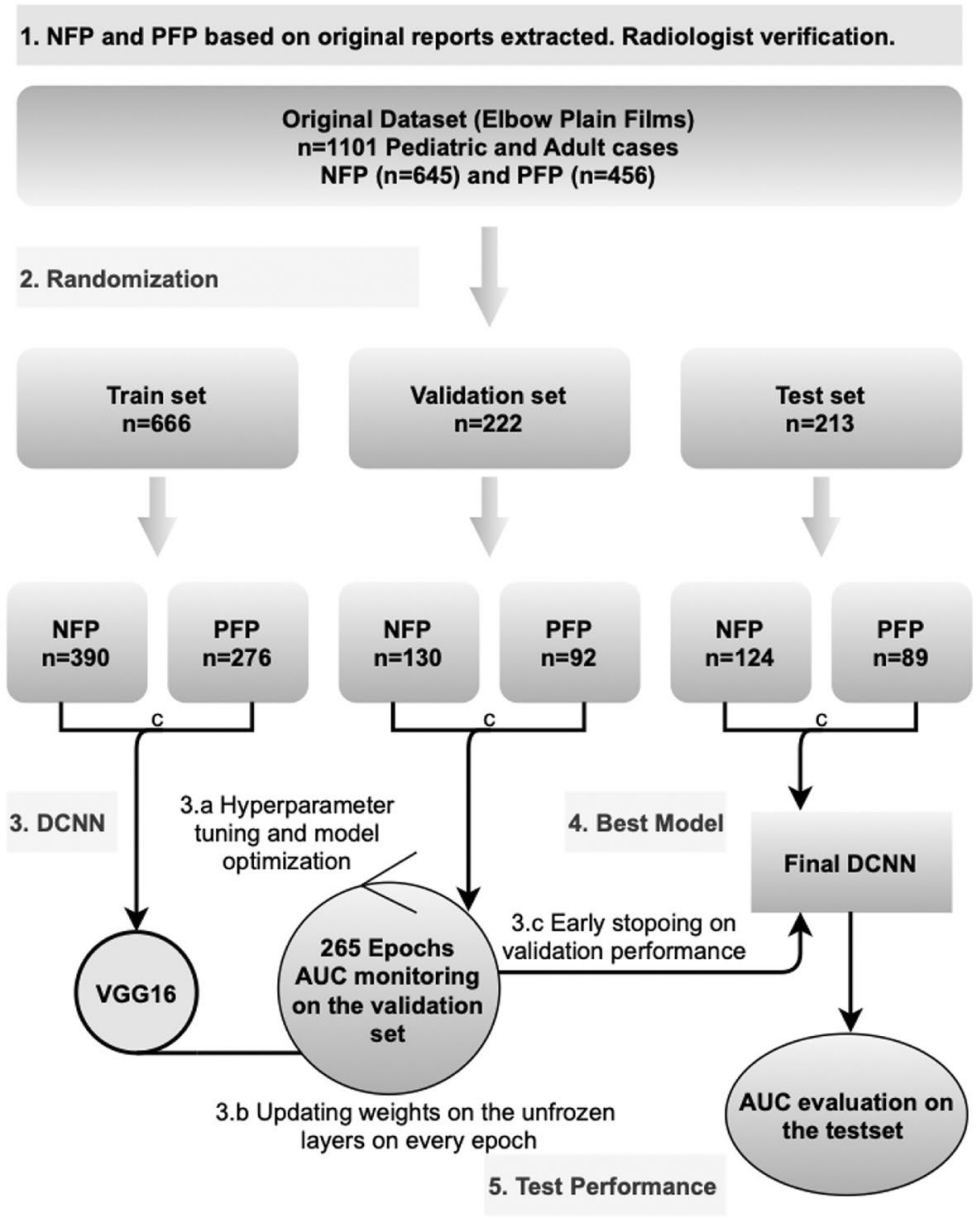
AI study method diagram. NFP = Negative Fat Pads/joint effusion; PFP = Positive Fat Pads/joint effusion; DCNN = Deep Convolutional Neural Network; ROC = Receiver Operating Characteristic; AUC = Area Under the Curve.

Cases were selected and separated to either positive joint effusion/fat pad group (n = 645) or negative joint effusion/fat pad group (n = 455) (Fig. 2). Cases were selected so that they indicated the presence of either positive or negative joint effusion in radiographs based on radiologic reports. Inclusion criteria were (a) history of recent trauma, (b) adequate radiographs (LAT projection fills the good radiographic criteria), and (c) the radiology report stating the presence or absence of joint effusion. There were several dropped cases due to LAT projection not meeting the good radiographic criteria in test set (n = 5), train set (n = 10) and in validation set (n = 2). Exclusion criteria were (a) metal objects (e.g., surgical hardware) in the field of view, (b) dislocation of the elbow joint, (c) comminuted fracture of the elbow, and (d) control study of previous trauma. In addition, to improve the accuracy of joint effusion cases were reviewed by an external reader who is qualified for MSK reporting (3 years experience). In cases where the consensus was met with the original radiology report they were included in the study. There were a number of cases excluded because of disagreement in the dataset (n = 32, 2.9%). Cases were randomly split into three subsets: train (n = 666), validation (n = 222), and test (n = 213).

In this study DCNN model performance was evaluated compared to three radiologists with 23, 29 and 21 years of clinical experience, respectively. Radiologists labeled test set lateral elbow radiographs in positive or negative joint effusion groups. This was then compared to DCNN model results. In addition, two data categories were created: 4-projection and lateral projection (Table 3). Each patient directory consisted of between 2 and 6 radiographs of different projections.

**Table 3:** The number of images in train, validation and test set in 4-projection and lateral dataset.

Data category	Train	Validation	Test
Four-projections	2672	892	859
Single lateral projection	944	229	215

### Image pre-processing

The dataset was pre-processed including conversion of the original radiographs in DICOM to Portable Network Graphics format and resized them to 224 × 224 pixel and 72 pixel/inch resolution. All images were rescaled by a factor of 1/255 for the pixel intensity normalization. In addition to image standardization, the train set images are augmented to prevent overfitting during training. The augmentation is done with random horizontal flips, random rotations of up to 40°, random width and height shifts of up to 0.2 (20%), random shear angling up to 0.2 (20%), and random zooms up to 0.2 (20%). The augmentation was implemented using the Keras' ImageDataGenerator framework which applied these image transformations along with the network training.

### DCNN model selection and classification

Multiple DCNNs were examined including VGG16^31^, MobileNet^32^, Residual Nets^33^, Inception Residual Net^34^, NASNet Large^35^, DenseNet^36^, and CheXnet^25^, with varying network architecture and hyperparameter settings as briefly describe in Table 4. Initially, these models were studied with different base pretrained architectures and depths. On the basis of the primary experiments, the VGG16^31^ was deemed to be the most reliable network for fine-tuning. All the DCNNs were initialized with the previously trained weights obtained from ImageNet dataset^37^.

**Table 4:** Different DCNN models trained in this study and model descriptions.

Model	Description	Number of layers/trainable parameters
VGG16	A 16-layer architecture consisting of convolution layers, Max-pooling layers, and 3 fully connected layers at the end. It has a deep network but end-to-end small 3 × 3 Convolutional filters	16 layers 138.4M parameters
DenseNet201	A CNN architecture consisting of Densely connected blocks, where each layer input comes from previous layer output feature maps. It has two block types, Dense blocks including batch normalization, ReLU activation and 3 × 3 convolution layers, a Transition layer consisting of Batch normalization, 1 × 1 convolution and Average pooling layers. Transition blocks are placed after each dense blocks	402 layers 20.2M parameters
MobileNet	An architecture that utilizes depth-wise separable convolutions and thus reducing the number of parameters. These are made of two operations: depthwise convolution for filtering, and point-wise convolution for combining the outputs of depth-wise convolutions with 1 × 1 convolution	55 layers 4.3M parameters
ResNet152	The main feature of ResNet architecture is the existence of residual blocks that utilize shortcuts to skip some layers. Each residual block consists of two Conv-layers, with batch normalization and ReLU activation, using 3 × 3 filters with stride 1. Resnet is famous for solving the Vanishing Gradient problem	307 layers 60.4M parameters
InceptionV3	A CNN model that is made of symmetric and asymmetric building blocks that consist of Convolutions, AVG-pooling, Max-pooling, dropouts, and fully connected layers. The convolutions are factorized that results in a reduced number of learnable parameters	189 layers 23.9M parameters
NASNetLarge	Stands for Neural Search Architecture network and works best on small datasets. In simple terms, it automates the network architecture engineering, and identifies and evaluates the performance of possible architecture designs without training. Furthermore, it utilizes a regularization technique called ScheduledDropPath	533 layers 88.9M parameters
CheXNet	It is a 121 layer Convolutional neural network that inputs a chest X-ray image and outputs the probability of a pathology	121 layers 6.9M parameters

Two models using VGG16 as the base architecture were trained as follows:

1. **Model A:** Training on the dataset containing lateral projection images only.
2. **Model B:** Training on the dataset containing all 4-projection images.

Both models considered the area under the receiver operating characteristic curve (AUC-ROC) as the classification metric for monitoring.

**Figure 3:**
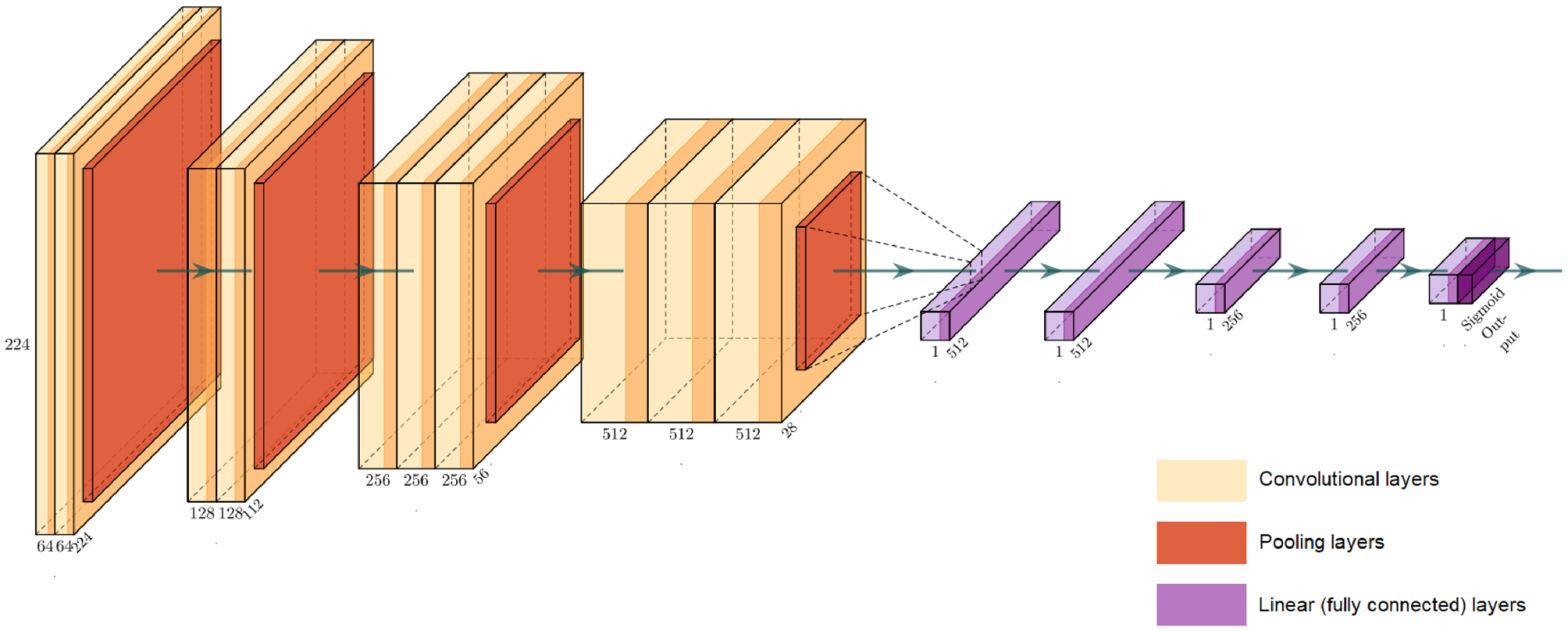
Architecture of the modified VGG16 model trained for this paper.

From the topless VGG16 model pre trained with a large collection (more than 14 million images) of human annotated images (ImageNet), the last convolutional block was chopped off, and four layers including batch normalization, max-pooling, flattening, and a fully connected neural network with a rectified linear activation (ReLU) function were added on top. The model architecture uses the same first 4 convolutional blocks as VGG16. Then the output of the last convolutional block is flattened and the following layers are added: 1. connected layer with 512 neurons, 2. connected layer with 512 neurons, 3. connected layer with 256 neurons, 4. connected layer with 256 neurons. Finally, an output dense layer using the Sigmoid activation function was added that produced prediction values between 0 and 1 corresponding to the prediction probabilities of the negative and positive classes, respectively. The base of the model was frozen, and the added layers were trained for 256 epochs using a learning rate of 1e-05, binary cross-entropy as the loss function, batch size of 32, and Adam as the optimizer. Early stopping call back was also used which stops the training process when there is no learning progress with the network, meaning that the neurons stop updating the weights to avoid overfitting the model. Figure 3 represents the modified VGG16 model trained for this paper. The network was fine-tuned by unfreezing the top layers of the frozen base model and was trained on both the newly added network layers and the last layers of the base model. This allowed us to fine-tune the higher-order feature representations in the base model to make them more relevant for the specific task. The experiments were performed on a virtual workstation with NVIDIA TITAN V100 GPU and 32 GB memory.

### Statistical analysis

The statistical analyses were performed in Python (version 3) and using a scikit-learn library (version 0.22.2)^37^. Accuracy, precision, recall, specificity, F1 Measure, and Cohen’s kappa coefficient were calculated to evaluate the performance of the deep learning models. Two-sided 95% confidence intervals (CI) were used for an aggregate measure of model performance and network stability, and to be more conservative for accuracy. McNemar’s Chi-Square significant test was used to compare paired predictions obtained by the neural network model and radiologist experts. The CI for the performance metrics was obtained with 10 replications of the entire train, validation, and test process. The convolutional neural network was trained using Keras (version 2.3.0) and TensorFlow (version 2.2.0).

To estimate the reliability and agreement of the expert radiologists, pairwise observer agreements were measured. Overall inter-rater agreement (Cohen kappa statistic) was calculated using the Pingouin package in Python programming language^39^.

## Results

**Figure 4:**
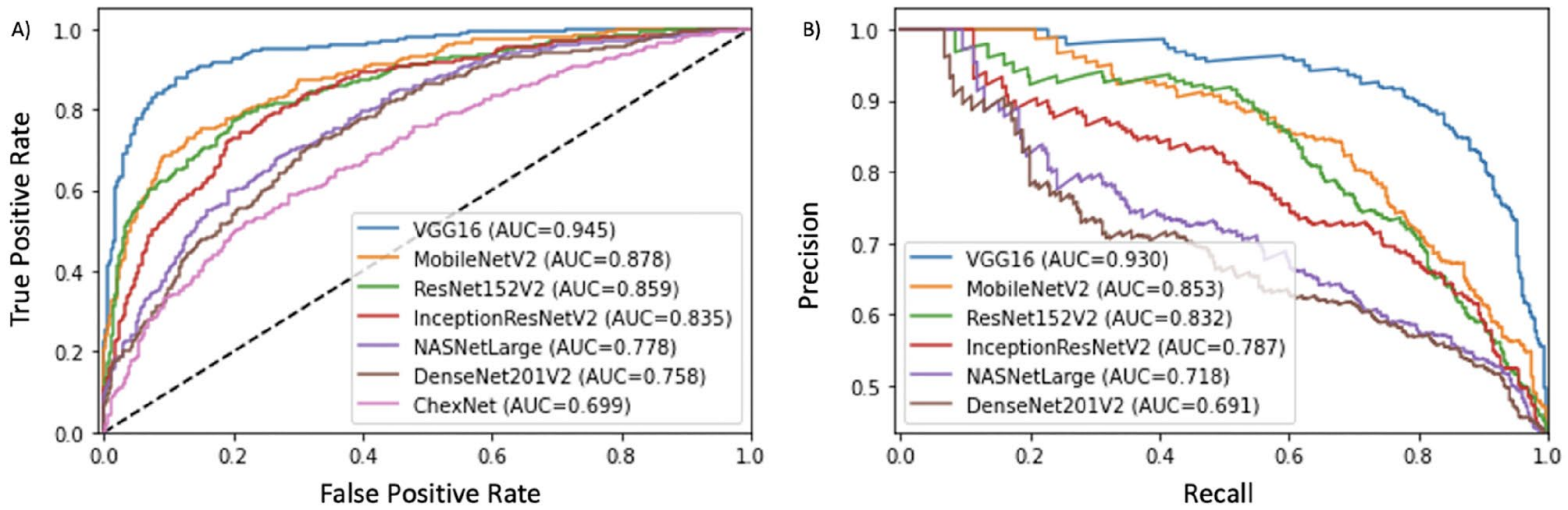
Different deep learning model comparisons are seen with their true positive rate (**A**) and precision (**B**).

Comparison of AUCs obtained from these models in this study is shown Fig. 4. Different DCNN models showed variation in ROC (Fig. 4A) and Precision-Recall (Fig. 4B) area under the curves ranging from 0.699 to 0.945, and 0.691 to 0.933, respectively.

**Figure 5:**
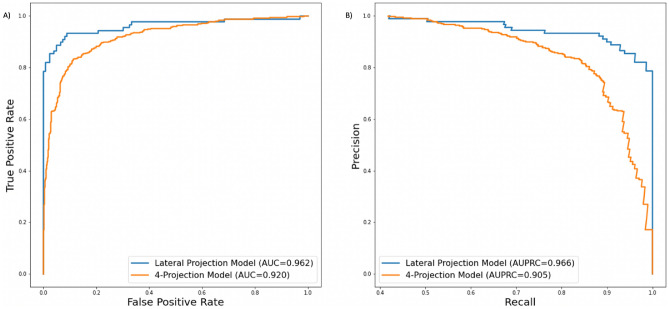
ROC AUC (**A**) and AUPRC (**B**) obtained from best training iterations for Models A (Lateral Projection data) and B (4-Projections data). AUC = Area Under the Curve and AUPRC = Area Under the Precision Recall Curve.

**Figure 6:**
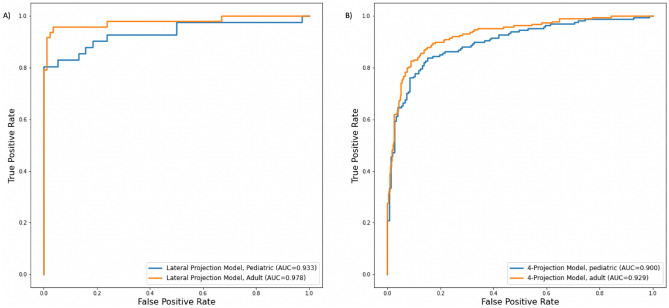
AUC curves obtained from best training iterations for Model A with lateral projection data (**A**) and Model B with 4-projections data (**B**) in pediatric and adult patients. AUC = Area Under the Curve.

ROC and Precision-Recall area under the curves obtained over the best training iteration for models A and B are shown in Fig. 5. The proposed deep learning framework showed an AUC of 0.951 (95% CI 0.94–0.955) and 0.906 (95% CI 0.89–0.91) for the model A and B in the test set, respectively. ROC curves obtained over the best iteration for adult and pediatric patient groups separately are shown in Fig. 6. The AUC’s of the radiologist experts in the test set showed a range of 0.923 to 0.928 with no significant statistical difference.

**Figure 7:**
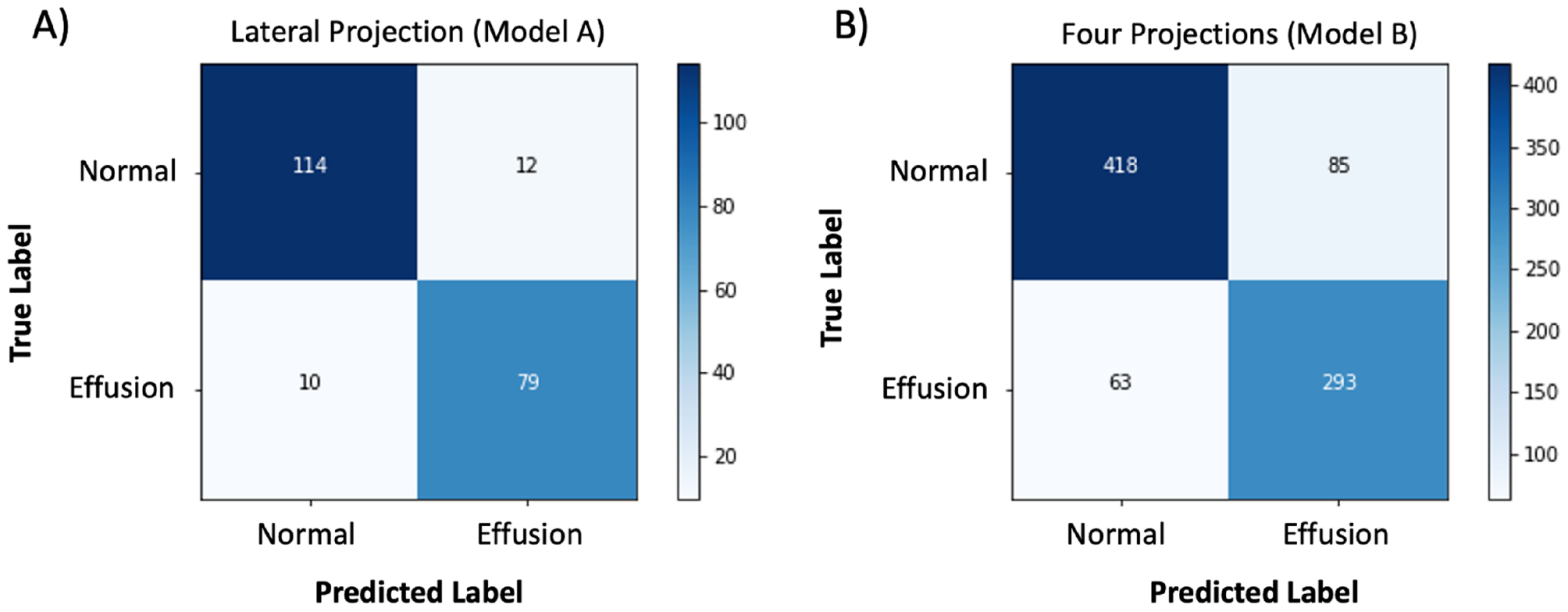
Averaged confusion matrices for the model A and B over the 10 iterations. Confusion matrices show performance of a deep learning model that was trained with only lateral projection (**A**) and a model that was trained with four projections (**B**).

The average confusion matrices of the models A and B are shown in Fig. 7. Table 5 shows the diagnostic performance of deep learning models for lateral projection and four projection views. The average accuracy, sensitivity, specificity, F1 score, and AUC of the model A and B including pediatric and adult patient groups separately are reported in Table 5.

**Table 5:** Classifications scores of Model A and Model B. Values (except F1-score and AUC) are shown in percentage. The Confidence level was set to 95%. (P) = Pediatric patients only, (A) = Adult patients only.

Model	Precision	Sensitivity	Specificity	Accuracy	F1-score	AUC
Model A	86.8% (83.3–90.3)	88.5% (87.0–90.1)	90.2% (87.1–93.3)	89.5% (88.0–91.0)	0.876 (0.86–0.89)	0.951 (0.94–0.96)
Model B	77.9% (75.1–80.8)	82.2% (76.8–87.5)	83.1% (79.4–86.7)	82.7% (81.8–83.6)	0.797 (0.78–0.81)	0.906 (0.89–0.91)
Model A, (P)	86.4% (83.7–89.1)	84.9% (82.4–87.3)	85.3% (81.7–88.9)	85.1% (83.6–86.5)	0.856 (0.84–0.87)	0.924 (0.91–0.93)
Model A, (A)	87.3% (82.7–91.8)	91.7% (89.9–93.4)	92.4% (89.2–95.6)	92.1% (90.2–94.0)	0.893 (0.87–0.92)	0.966 (0.96–0.97)
Model B, (P)	83.4% (81.0–85.7)	75.7% (73.0–78.3)	83.3% (80.1–86.4)	79.3% (77.7–81.0)	0.793 (0.78–0.81)	0.866 (0.85–0.88)
Model B, (A)	83.3% (80.4–86.2)	78.0% (74.5–81.5)	91.3% (89.1–93.6)	86.7% (85.8–87.5)	0.804 (0.79–0.82)	0.924 (0.92–0.93)

**Figure 8:**
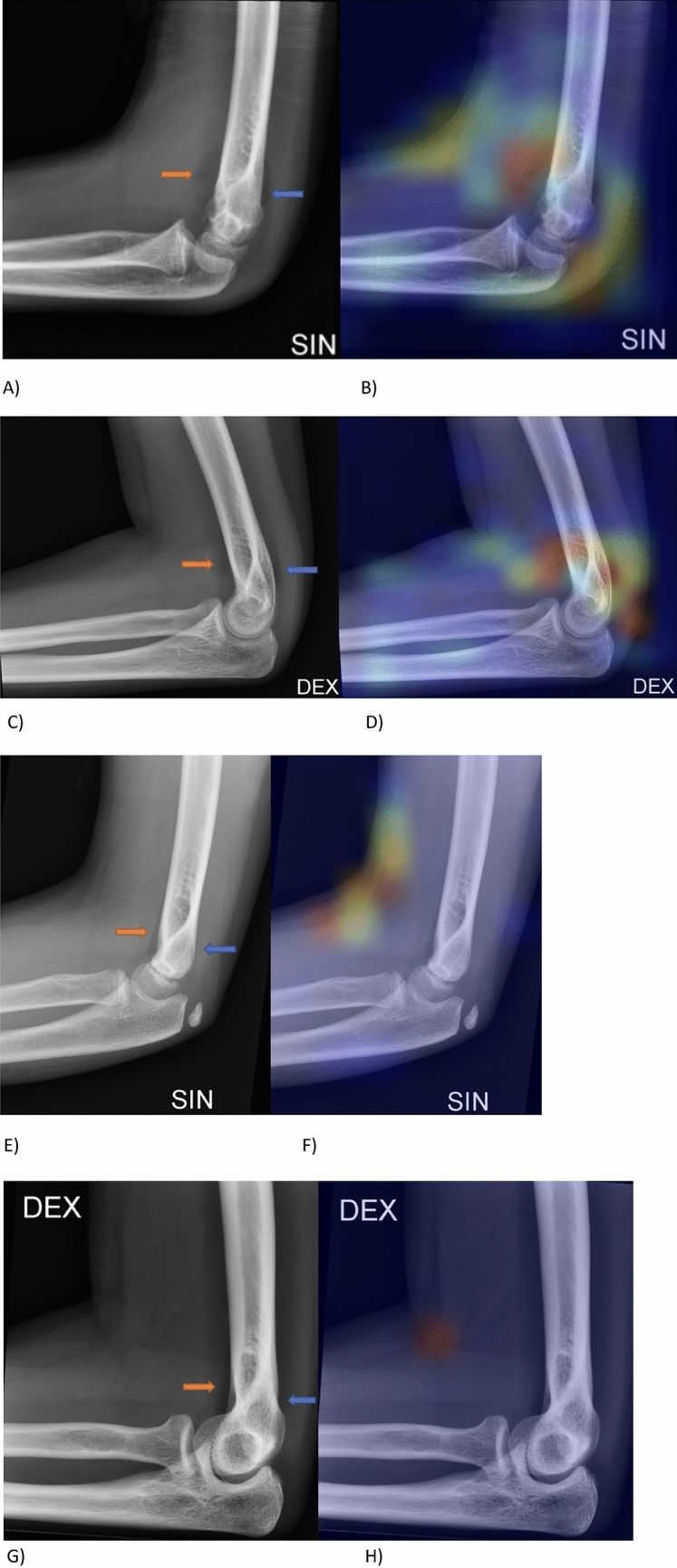
Pediatric (Male, 10y) and adult (Female, 19y) patient with joint effusion (**A**,**C**) seen anteriorly (orange arrow) and posteriorly (blue arrow) but without visible fracture. Heat map highlights joint effusion (**B**,**D**). Pediatric (Female, 8y) and adult (Female, 36y) patient with no joint effusion (**E**,**G**) anteriorly (orange arrow) or posteriorly (blue arrow) and without visible fracture. Heat map does not highlight normal joint effusion (**F**,**H**).

In elbow joint effusion classification with DCNN activation heat map visualizations were obtained using Keras library Grad-CAM class. Examples of these heat map visualizations are shown in Fig. 8. Grad-CAM class allows delineating a heatmap that highlights areas of the image that the neural network was able to extract important features of positive and negative joint effusion classes. Looking at the Grad-CAM activation visualization it can be noted that when there is joint effusion present in radiographs it concentrates on the anterior and posterior joint effusion regions (Fig. 8A–D). On the contrary, when there is no joint effusion present in radiographs the heat map does not highlight these anatomical regions (Fig. 8E–H). The normal undisguised anterior joint effusion did not show heat map visualization (Fig. 8F,H).

In this study three radiologists labeled cases based on joint effusion appearance in test set lateral radiographs. Radiologists’ classification performances and AUCs are reported in Table 6. Compared with the AI model A at the average operating point, the three radiologists showed an average accuracy, sensitivity, specificity, precision, F1 score, and AUC of 92.8%, 91.7%, 93.6%, 91.07, 91.4%, and 92.6, respectively. The judgments provided by the three radiologists showed substantial agreement. The overall Cohen Kappa agreement between the three reviewers was 0.801 (0.779–0.827). There was no statistically significant difference between the AI model A and the three radiologists in elbow joint effusion classification (*p* value > 0.05).

**Table 6:** AI model and Radiologist performance comparisons on the lateral elbow test set.

Model	Model A	Radiologist 1	Radiologist 2	Radiologist 3
AUC	0.951	0.927	0.923	0.928
Accuracy	89.8%	93%	92.5%	93%
Sensitivity	88.8%	92.1%	91.01%	92.1%
Specificity	90.5%	93.6%	93.6%	93.6%
Precision	86.8%	91.1%	91.01%	91.1%
F1 score	87.8%	91.6%	91.01%	91.6%

## Discussion

In this study it was demonstrated that DCNN can classify elbow joint effusion in pediatric and adult patients with an average AUC of 0.95. Especially in the elbow there can be occult fractures that are not visible in radiographs^1,2^ and joint effusion is an important finding^7^ determining patient’s treatment. In addition, due to the importance of joint effusion in the radiographs the developed DCNN can be very helpful to radiologists, radiology trainees or general practitioners to highlight this important finding. Deep learning algorithms that can accurately, reliably, and rapidly classify radiological images into normal and pathological findings have considerable clinical value, because they can lessen the burden on both the radiologist and the referring physician by providing fast automated diagnostics. In such cases DCNN presented in this study could have clinically significant impact on patient management. The developed model showed good sensitivity, specificity and accuracy for elbow joint effusion classification and differentiation.

England et al.^28^ demonstrated that DCNN can accurately detect elbow joint effusion from lateral projections in pediatric patients. Our study considered a less complex artificial neural network (VGG16) with only 16-layers in joint effusion classification and included both pediatric and adult patients. This approach is more general as there is more anatomic variation when both adults and pediatric patients are included on model training. Pediatric elbow joint is different compared to adults and not least because of the ossification centers. In addition, joint effusion classification can differ from adults because undeveloped coronoid fossa and olecranon fossa and soft tissue injury might result in appearance of joint effusion^40^. DCNN showed slightly better AUC for lateral view in adult patients compared to pediatric patients 0.966 and 0.924, respectively. The difference is small and might be related to above mentioned anatomic differences, but also to the smaller sample size of pediatric patients in model training. Increasing the amount of pediatric cases in training might bring the models performance with pediatric patients closer to the performance with adults.

AI has reached the level of radiologists in elbow radiographs^29,30^ and in other regions^22,23^. In this study, AI performance was compared to three expert radiologists to whom it was superior in AUC comparison. Compared to PGY5 emergency medicine residents in a previous study^28^ AI in this study showed higher specificity but lower sensitivity and accuracy.

To our knowledge, there is a limited number of studies that utilize multiple radiographic projections using DCNN^30^ which is more difficult and time consuming due to anatomic variation. Joint effusion is clinically evaluated on lateral projections, but it can be seen in other elbow projections including radial head projection. Results in this study showed that using only the lateral projection in effusion classification was superior to using 4 projections in AI approach. Model A which was trained on the lateral view resulted in higher AUC as compared to the model B which was trained with all projections. This indicates that when training with the lateral projection images, the CNN model better extracts high level features representing the joint effusion, as expected. A comparison of the two models reveals superior classification performances for the model A in average, but the results obtained for the model B still support the feasibility of determining effusion from other projections using AI as well. In future studies, however, it may be interesting to see if using more or different projections adds sensitivity or accuracy. The approach used in this study’s deep learning model can be further developed to extend imaging findings from joint effusion to other critical findings where all projections are necessary^2^.

Findings in this study indicate that a lateral projection approach yields best results. One interesting future direction to further improve the performance of DCNN is to include vision transformers (ViT) and generative adversarial networks (GAN) to generate radiographs without any need for subject-specific labeling. This can be obtained by learning to generate images that imitate the patient’s musculoskeletal features and elbow joint characteristics which are learned in an unsupervised manner from x-ray images of the positive and negative cases.

### Limitations

In this study there are several limitations. First, this study was a retrospective single center study without external dataset which may affect the generalization of the results. Second, our dataset could have been larger, and AI was tested with one hospital data only. Therefore, our model may not be generalized enough for the larger population. In addition, this model was investigated on data from one x-ray device, and it would be beneficial to test the model with multicenter study^36^. Third, joint effusions were classified based on consensus with the radiologist's report and external validation which are subjective assessments. Further validating joint effusion with MRI would have been more objective. While it was not possible in the present study setting, it would add an objective gold standard to the assessment. Without such correlation, as in most clinical situations, it is usually the radiologist's report which determines the diagnosis and directs care of the patient. Binary classification could have been made using the three radiologists’ evaluations to obtain a stronger agreement on the labeling process prior to training the DCNN, but this was used in post validation to see if there were inter-radiologists' variations and if there were agreement between the AI model predictions and radiologists’ prediction. Finally, in the future it could be beneficial to use other DCNN models and compare the performances in joint effusion classification.

## Conclusion

In this study an automated method based on transfer learning was developed to classify joint effusion from elbow radiographs at a level comparable to a radiologist. DCNN classified joint effusions in both pediatric and adult patients with high accuracy. With AI-assisted interpretation of radiograph images at the level of experts, we hope that this technology can enhance the status of the radiology delivery, and patient's treatment.

## Data Availability

The datasets generated and/or analysed during the current study are not publicly available due to IRB restrictions but are available from the corresponding author on reasonable request.
